# Germination ecology and environmental influences on prickly golden fleece (*Urospermum picroides*) and implications for weed management

**DOI:** 10.1038/s41598-025-17501-4

**Published:** 2025-09-01

**Authors:** Ahmad Zare, Elham Elahifard

**Affiliations:** Department of Plant Production and Genetics, Faculty of Agriculture, Agricultural Sciences and Natural Resources University of Khuzestan, Bavi, Mollasani, Iran

**Keywords:** Burial depth, Cutting time, Growth hormone, Osmotic potential, Salinity, Plant ecology, Ecology, Plant sciences

## Abstract

Knowledge of the germination ecology of weed species provides information about their potential aggressiveness and helps develop effective weed management strategies. Therefore, the influence of gibberellic acid (GA_3_) and environmental factors (temperature, light, osmotic stress, salinity, cutting times, and seed burial depth) was evaluated on seed germination and seedling emergence of *Urospermum picroides a winter annual weed*. The results indicated that maximum seed germination was 94% and 83% when seeds were soaked for 12 and 24 h with 1000 and 800 ppm of GA_3_, respectively. Seed germination was not influenced by light conditions but was influenced by temperature. The highest germination percentages (95% and 93%) occurred at a constant temperature of 30 °C and an alternating temperature of 20/10°C, respectively. In response to light, the results showed that *U. picroides* is non-photoblastic and can germinate in darkness. Seed germination in response to different cutting times demonstrated that maximum germination was observed in brown achenes (81%), while minimum germination was obtained in white (0%) and yellow (4%) achene stages. Seed germination decreased from 92.5 to 12.5% as water potentials decreased from 0 to -0.4 MPa, and germination was completely inhibited at -0.5 MPa. The salt concentration required for a 50% reduction in maximum germination was estimated at 170 mM NaCl. Maximum seedling emergence occurred at an optimal burial depth of 1.18 cm. In conclusion, this study indicated that at lower soil depths, *U. picroides* is likely more fit than other species under conditions of low to moderate water and saline stress.

## Introduction

Prickly golden fleece (*Urospermum picroides* (L.) Scop. ex F. W. Schmidt) is an annual herb belonging to the family Asteraceae. It possesses edible, medicinal, and ecological values^1–6^. Notably, Consumption of this plant has been associated with a reduction in postprandial platelet aggregation in individuals with metabolic syndrome^7^. In addition to its functional uses, *U. picroides* has been identified as an weed in various regions, including Egyptian agricultural systems, Cyprus, and southwestern Australia, where it competes with native vegetation and cultivated crops^8–10^. Based on field monitoring, this weed is abundant in farmalnds, landscapes, and green spaces of Khuzestan, Iran.

Germination and seedling establishment are pivotal processes for expanding the ecological amplitude and distribution of plant species within agroecosystems^11^. In particular, seed germination represents a crucial phase that determines the success and dominance of annual weeds within their ecological niches^12^. Seed germination is influenced by various environmental and agronomic factors, which are critical for its success^13^. Key determinants of seed germination include osmotic potential, salinity, soil pH, the composition of the soil solution, temperature, and both the quality and intensity of light^14–19^. In addition, agricultural practices, maternal plant growth conditions, seed burial depth resulting from tillage, straw residue in the field, photoperiod, and hormonal regulation exert significant influence on seed germination^18,20–23^. Seed dormancy is a complex physiological process regulated by both environmental cues and endogenous mechanisms. The most important endogenous factors controlling seed dormancy and germination are plant hormones such as gibberellins, especially GA_3_^24^. Among the factors discussed, temperature and soil moisture emerge as the most critical determinants^25,26^as seed germination begins with water uptake under favorable conditions^27^.

The presence of this weed in urban green spaces poses a persistent challenge to ecological management. Among the available control strategies, cutting annual weeds is particularly effective in preventing reproductive success. However, determining the optimal timing of cutting is critical to inhibit seed germination from immature fruits and to ensure the long-term efficacy of the management approach^21,28^.

Climate change has exacerbated challenges in weed management by profoundly altering weed–crop interactions, growth dynamics, species composition, and land-use patterns. Although cultural, mechanical, biological, and chemical control strategies are typically tailored to specific weed species, cropping systems, and crop types, their effectiveness is increasingly undermined by climate-induced disruptions^29^. Elevated temperatures have exacerbated salinity and drought conditions, particularly in warmer regions. Consequently, weed seeds are exposed to varying temperature and moisture levels, leading to potentially inconsistent germination patterns across different soil depths^12,30^.

A comprehensive understanding of weed biology is essential for the success of integrated weed management (IWM) programs, as it enables informed, strategic decision-making for effective weed control and long-term management^31^. Moreover, such knowledge is critical for predicting the potential spread of weeds into new habitats. In particular, detailed insights into seed germination and seedling emergence are essential for developing effective and sustainable weed management strategies^32^ .

Currently, there is a notable paucity of information regarding the biology of *Urospermum picroides*, particularly in relation to its dormancy mechanisms, germination behavior, and seedling emergence under diverse environmental conditions. Addressing this knowledge gap is essential for advancing our ecological understanding and for developing targeted, effective management strategies for this species. Accordingly, the present study aimed to (a) evaluate the role of gibberellic acid (GA_3_) in breaking seed dormancy, and (b) investigate the effects of key environmental factors including temperature, light, salinity, drought stress, cutting time, and seed burial depth—on seed germination and seedling emergence of *U. picroides*, a winter annual weed.

## Results and discussion

### Effect of GA_3_ on breaking seed dormancy

**Fig. 1:**
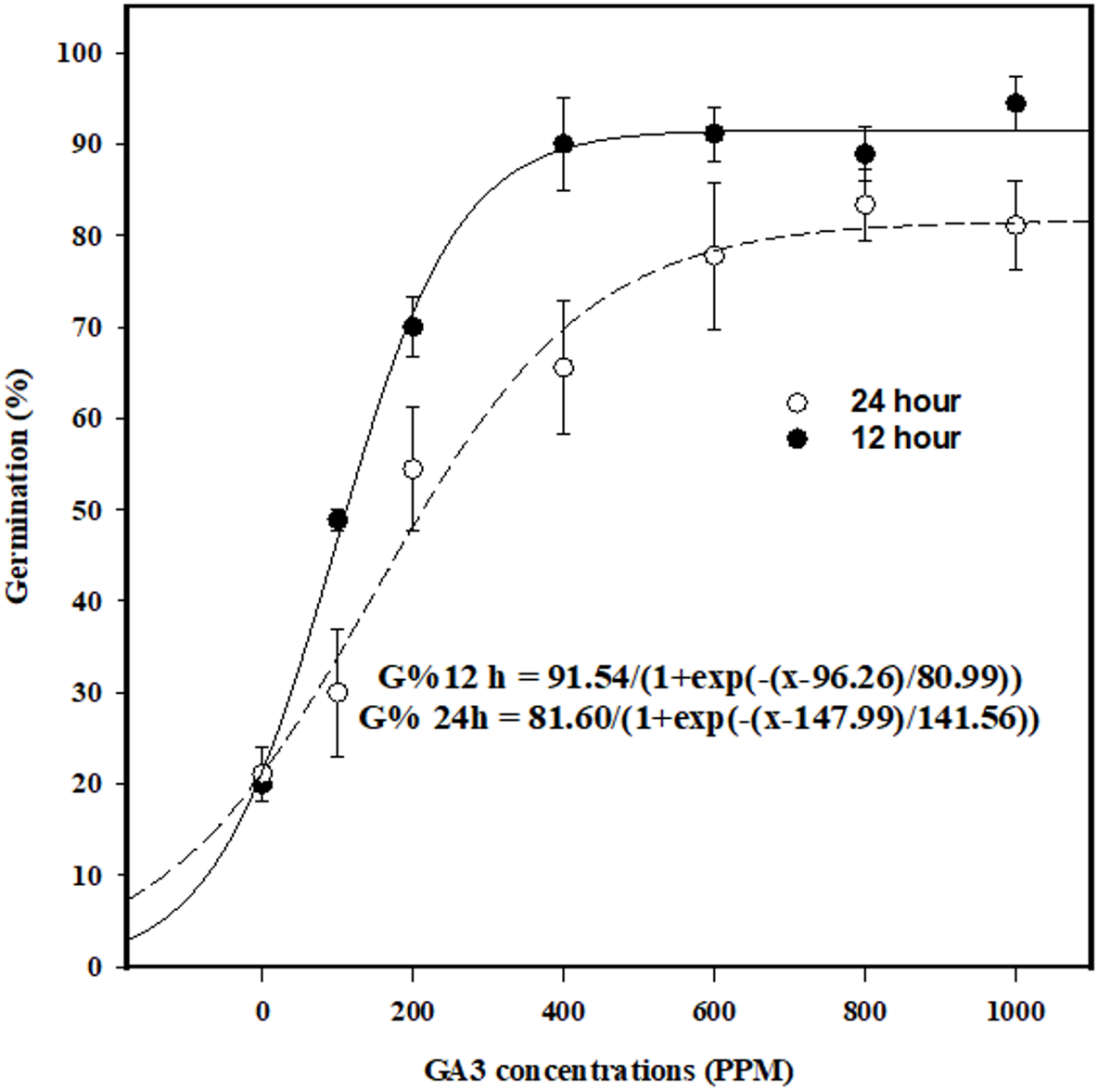
Effect of gibberellic acid (GA_3_) on seed germination of *Urospermum picroides* at 12 and 24 h seed soaking times. Vertical bars represent the mean standard error.

The seed dormancy experiment revealed that, in the absence of GA_3_, seed germination of *U. picroides* remained below 20%, with approximately 80% of seeds exhibiting dormancy. This suggests that seeds of *U. picroides* tend to remain dormant after rainfall from the parent plants. Maximum seed germination of 94% and 83% was achieved after 12 and 24 h of soaking with GA_3_ at concentrations of 1000 ppm and 800 ppm, respectively (Fig. 1). A sigmoidal model estimated that GA_3_ concentrations of 96 ppm and 147 ppm were required to reach 50% of maximum germination after 12 and 24 h of soaking, respectively (Fig. 1). Increasing GA_3_ concentration from 0 to 1000 ppm resulted in seed germination rising from 20 to 94% for 12 h of soaking, and from 21 to 81% for 24 h of soaking. Notably, seed germination plateaued beyond 400 ppm GA_3_ for 12 h soaking, with a similar trend observed at 600 ppm for 24 h of soaking.

Seed germination and essential plant functions require energy and nutrients^33^primarily supplied through the catabolic mobilization of seed storage reserves proteins, starch, and fat into soluble forms such as sugars and amino acids. This metabolic conversion is vital for supporting the growth and metabolism of the developing embryo^34^. The application of GA_3_ is a key strategy for breaking seed dormancy particularly in physiologically dormant species GA_3_ promotes germination by accelerating the mobilization of food reserves, modifying amino acid and protein synthesis, and suppressing the activity of growth-inhibiting hormones such as abscisic acid (ABA). This process involves regulating the hormonal balance within the embryo, shifting it from an inhibitory to a promotive state, thereby activating the metabolic and cellular mechanisms required for germination^35,36^. During germination, gibberellins biosynthesized by plant embryos induce de novo synthesis of the hydrolytic enzymes α-amylase and β-amylase. These enzymes mobilize stored carbohydrates within seed endospermic reserves, thereby enhancing enzymatic accumulation in viable seeds to sustain embryonic growth^37^.

It is noteworthy that both soaking duration and gibberellic acid (GA_3_) concentrations significantly influence the efficacy of dormancy-breaking treatments, with responses varying across weed species. For example, in *Brassica tournefortii*, seed germination exceeded 88% when treated with GA_3_ at 100 mg kg^−1^, whereas higher concentrations such as 500 mg kg^−1^ markedly reduced germination to 12%^38^. This finding highlights the efficacy of GA_3_ in stimulating dormant seeds to germinate. Additionally, following seedling emergence, the strategic use of herbicides or tillage offers effective approaches for managing weed populations^38^. The application of GA_3_ at concentrations of 200, 400, 600, and 800 ppm significantly enhanced the germination of *Erechtites hieraciifolius* seeds, with germination exceeding 95%. In contrast, the control treatment exhibited seed germination of less than 20%^39^. The germination of *Papaver dubium* L. and *P. rhoeas* seeds treated with GA_3_ was significantly higher compared to the control treatment. However, the two populations exhibited distinct responses to GA_3_ concentrations. Maximum seed germination was observed at 750 ppm GA_3_ for *P. rhoeas* and at 500 ppm GA_3_ for *P. dubium*^40^. Previous studies have demonstrated that the application of GA_3_ significantly enhances seed germination^41–45^. GA_3_ has been proposed as an alternative treatemnt for promoting the germination of seeds that require light, low temperatures, or long-day conditions^46–48^.

### Effect of temperature and light on seed germination

**Fig. 2:**
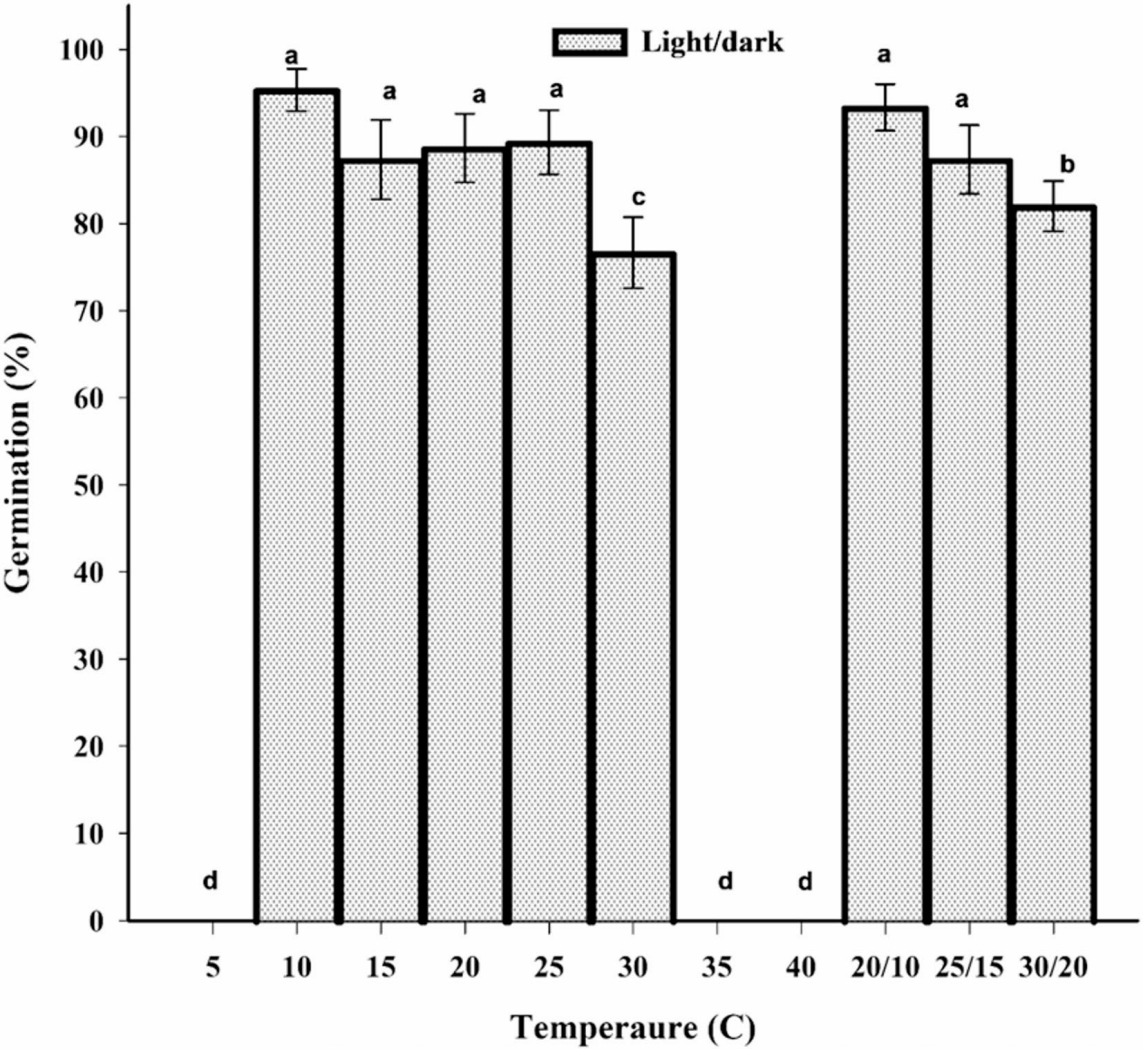
Effect of constant temperatures (light/dark) on seed germination of *Urospermun picroides*. Vertical bars represent the standard error of the mean.

ANOVA analysis indicated no significant interaction between temperature and light on the germination of *U. picroides* seeds (data not shown). germination was not affected by light conditions but was significantly influenced by temperature. The highest germination percentage (95%) was recorded at a constant temperature of 10 °C, which was significantly higher than germination at other constant temperatures, including 15 °C (87%), 20 °C (88%), 25 °C (89%), and 30 °C (76%). Notably, a statistically significant difference was observed only between 30 °C and the other constant temperatures (Fig. 2). Seed germination under alternating temperature conditions was significantly lower at 30/20°C (82%) compared to 25/15°C (87%) and 20/10°C (93%) (Fig. 2), no germination was observed at temperatures of 5, 35, and 40 °C under both light treatments. The maximum differences in germination percentage between constant and alternating temperature regimes were 19% and 11%, respectively. The optimal temperature for germination rate was 25 °C (data not shown).

Temperature governs seed germination through three principal mechanisms: (i) defining germination capacity and kinetics, (ii) releasing primary or secondary dormancy, and (iii) inducing secondary dormancy. Critically, temperature-mediated germination responses significantly influence population ecology, with broader thermal tolerance ranges correlating strongly with expanded geographic distributions in plant species^49^. Given that supra-optimal temperatures suppress germination more profoundly than suboptimal temperatures, this indicates that *U. picroides* populations likely present diminished agronomic challenges during warmer seasons, where reduced germination percentage (GP) is expected due to thermal inhibition. Consequently, the species exhibits reduced competitive ability and lower seedling establishment probability under transient heat stress events^50^. This study indicates that the optimal temperature range for germination of *U. picroides* is between 10 and 30 °C. Based on meteorological data from Khuzestan province, maximum germination is expected between December and February, when ambient temperatures remain below 30 °C. As temperatures rise in March, seed germination declines likely due to thermal inhibition. This study confirms that *U. picroides* is non-photoblastic, meaning it does not require light exposure to initiate germination. As a result, this species can successfully establish itself in urban green spaces, particularly beneath tree canopies.

Understanding the seed germination patterns of dominant weed species in a cropping region is vital for predicting weed seedling emergence. The predictability of emergence is essential for enhancing farmers weed management decisions^51^. The false seedbed technique leverages seed germination biology to deplete weed seed banks. Consequently, the efficacy of this management strategy depends critically on factors governing weed seed germination and seedling establishment. Key abiotic regulators including soil thermal regimes (mean temperature and diurnal fluctuations), moisture availability, light quality, soil nitrate concentrations, and the gaseous composition collectively modulate germination-to-emergence transitions in weed populations^51,52^. Beyond the false seedbed technique for seed bank reduction, altering planting dates particularly for ornamental flowers in urban green spaces, can constrain the competitive capacity of this weed species. In general, seed germination in weed species may be restricted to a specific temperature range, while some species exhibit the ability to germinate across a wider thermal spectrum^53^. The optimal germination temperature for *Sophora alopecuroides*, *Parthenium hysterophorus*, *Polygonum perfoliatum* L., and *Notobasis syriaca* has been determined to be 20 °C^19,54–56^.

In comparison to our findings, several studies have reported that certain weed species, including *Marrubium vulgare* L^57^. *Lactuca serriola*^58^*Mimosa pudica* L^59^. *Agrostemma githago*^50^ and *Sophora alopecuroides*^54^exhibit non-photoblastic germination. Conversely, other weed species, such as *Parthenium hysterophorus*^55^*Eclipta prostrata*^60^*Dinebra panicea* var. brachiata^61^*Picnomon acarna*^62^*Echium plantagineum*^63^and *Bidens pilosa*^64^are photoblastic, and light is critical to reach maximum seed germination.

### Effect of cutting time on seed germination

**Fig. 3:**
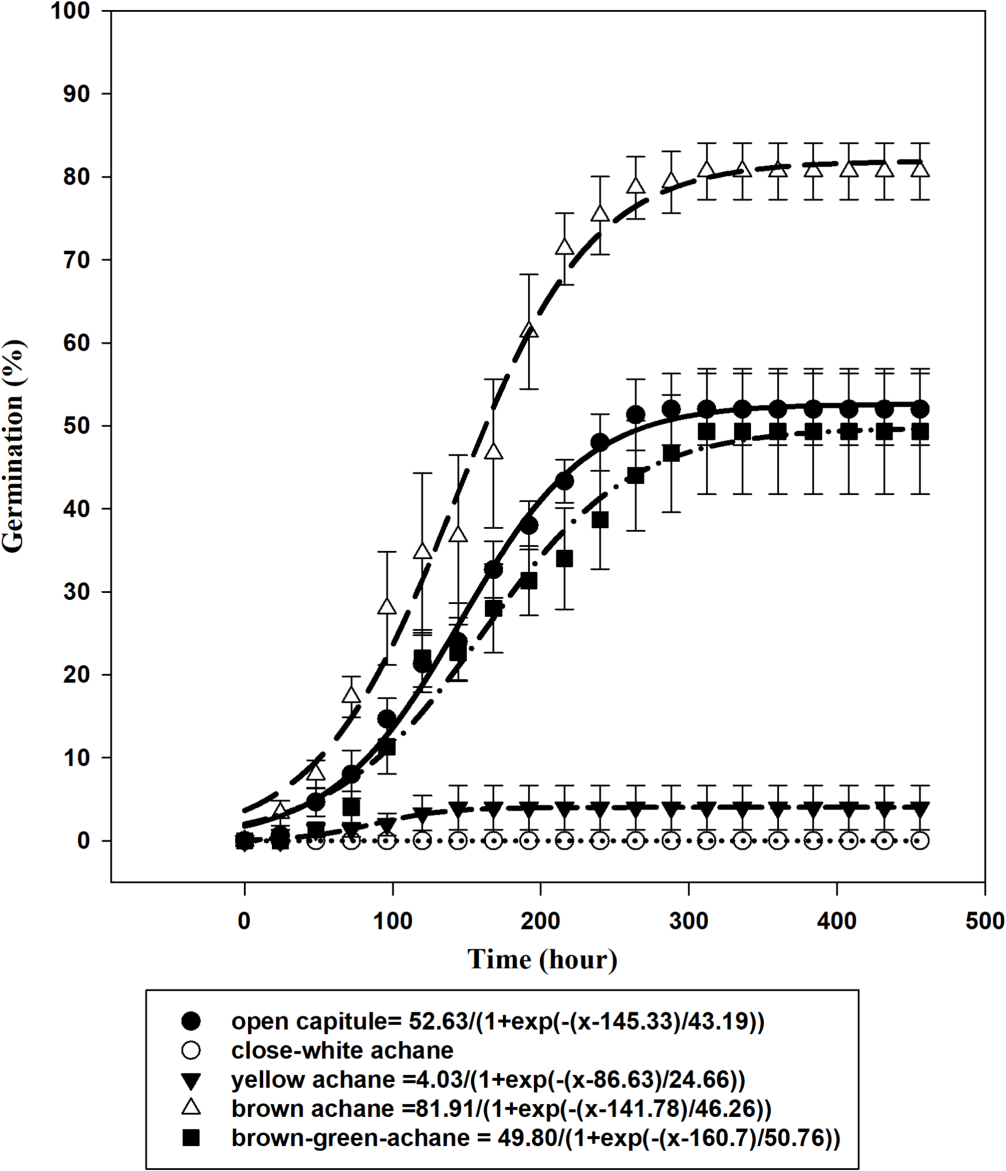
Effect of cutting time on seed germination of *Urospermum picroides*. Vertical bars represent the mean standard error.

Seed germination in *U. picroides* varied with cutting times, brown achenes exhibitined the highest seed germination (D: 81%). In contrast, minimal seed germination was observed in white (A: 0%) and yellow (B: 4%) achenes. A notable finding of this study was the relatively high germination rate (49%) observed in greenish-brown achenes (C). Moreover, seeds from open capitula exhibited significantly lower germination percentages compared to those from closed capitula containing brown achenes (Fig. 3). The maximum observed difference in germination percentage between open and closed capitula was 29% (Fig. 3).

The lack of seed maturation, particularly during the white and yellow stages of achene develompent, suggests that cutting time plays a critical role in seed viability. Premature cutting during early achene formation results in non-viable seeds with negligible germination potential. Furthermore, the findings suggest that during achene formation, particularly at the greenish-brown stage, approximately 50% of the seeds can germinate. However, an inappropriate cutting time may lead to contamination and increase the soil seed bank of weeds.

Using a sigmoidal model, the estimated time required to achieve 50% of maximum germination (T_50_ parameter) varied across achene types and capitulum conditions. Specifically, T₅₀ values were 145 h for seeds from open capitula (E), 86 h for yellow achenes (B), 141 h for greenish-brown achenes (C), and 160 h for mature brown achenes (D).

**Fig. 4:**
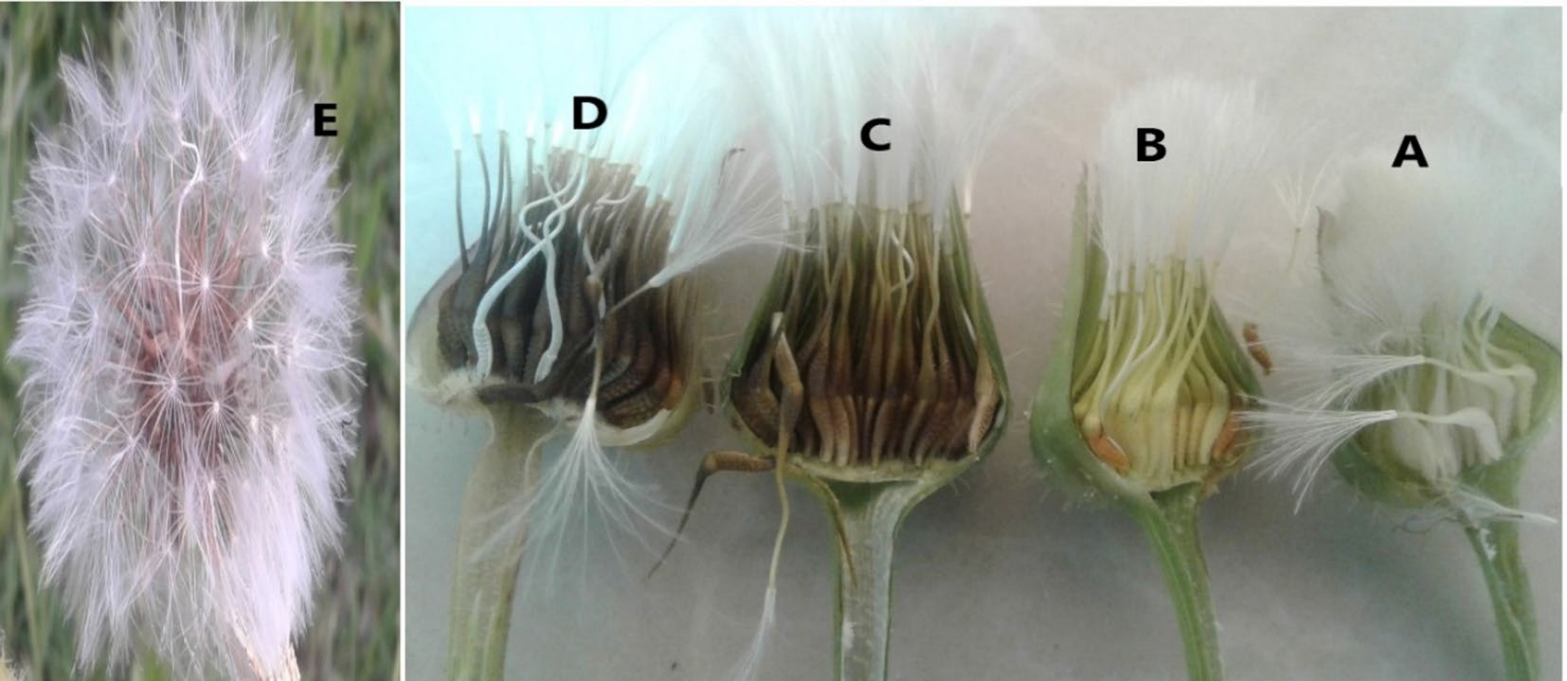
Seed maturation stages of *Urospermum picroides*: Closed capitule with white achene (**A**), closed capitule with yellow achene (**B**), closed capitule with greenish-brown achene (**C**), closed capitule with brown achene (**D**) and open capitule with brown achene (**E**).

The low or absent seed germination percentage at stages A and B (Fig. 4) is attributed to seed immaturity, likely due to incomplete synthesis of storage proteins essential for germination^65^. This aligns with findings in *Rumex crispus*, where the timing of seed harvest measured in days post-anthesis significantly influenced germination outcomes. Seeds harvested between 18 and 42 days after anthesis exhibited high germination rates (96–100%), whereas those collected at 6 days post-anthesis failed to germinate, indicating physiological immaturity^66^.

The timing of cutting significantly influenced seed germination in various Asteraceae weed species. Germination rates were markedly lower when plants were cut at the flowering stage compared to the dead-ripe stage. Seed germination responses varied markedly among Asteraceae weed species depending on the timing of cutting. At the dead-ripe stage, germination rates were high for most species: *Senecio vulgaris* L. (90%), *Aster tripolium* L. (90%), *Taraxacum vulgare* Schrank. (91%), *Hypochaeris radicata* L. (90%), and *Cirsium arvense* Scop. (38%). In contrast, cutting at the flowering stage significantly reduced germination, with rates dropping to 35%, 86%, 0%, 0%, and 0%, respectively^67^. Achene germination in *Senecio jacobaea* L. and *Carduus nutans* L. was significantly influenced by capitulum maturity. Germination was negligible (< 1%) in greenish achenes, indicating physiological immaturity. In contrast, greenish-brown achenes exhibited moderate germination rates (56–64%), while brown-hard achenes showed the highest viability (77–88%) across three study sites^68^.

### Effect of osmotic potential on seed germination

**Fig. 5:**
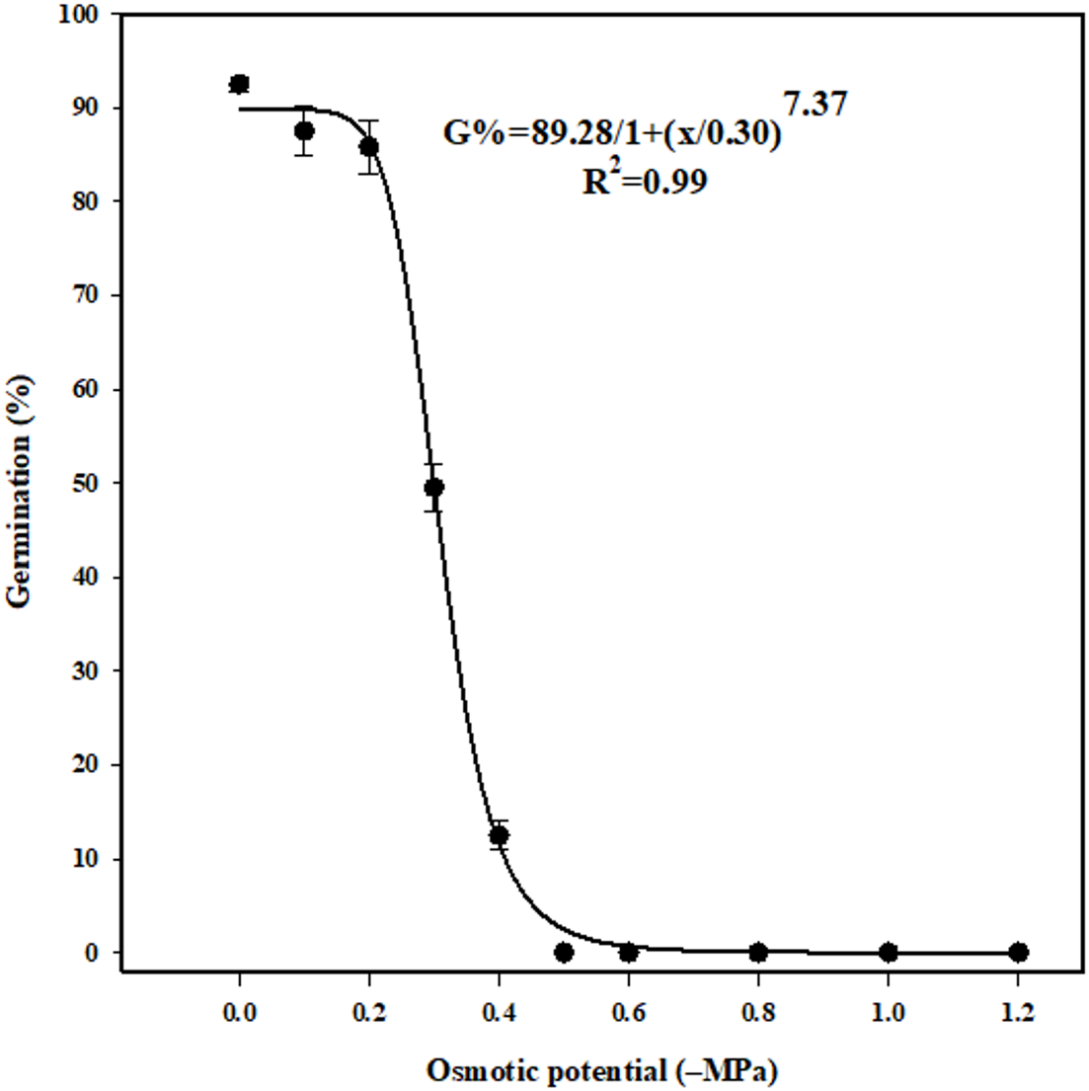
Effect of osmotic potentials on germination of *Urospermum picroides*. Vertical bars represent the mean standard error.

Seed germination of *U. picroides* declined from 92.5 to 12.5% as osmotic potential decreased from 0 to -0.4 MPa, with complete inhibition observed at -0.5 MPa (Fig. 5). Germination of incubated seeds at -0.1 and − 0.2 MPa osmotic potentials was 87% and 85%, respectively. Compared to the control treatment (non-stress), seed germination decreased by 5.5%, 7.5%, 46%, and 86% at osmotic potentials of -0.1, -0.2, -0.3, and − 0.4 MPa, respectively (Fig. 5). The osmotic potential required for 50% inhibition of maximum germination was − 0.30 MPa. An 85% germination rate at an osmotic potential of -0.2 MPa suggests that *U. picroides* seeds can germinate under mild water-stress conditions. Given this species’ ability to germinate under low drought stress and its optimal germination within a temperature range of 10–30 °C, seed germination likely coincides with winter precipitation.

Drought stress is a major abiotic constraint affecting weed seed germination. This research demonstrates that insufficient moisture significantly reduces seed germination, highlighting the susceptibility of *U. picroides* to drought conditions. As a result, its establishment is more likely in moisture-rich soils compared to areas with limited rainfall. Under drought stress, seedlings emerge weaker, reducing their competitive ability and ultimately compromising survival and seed reproduction. Moreover, seeds subjected to osmotic stress struggle to attain adequate moisture for imbibition due to restricted water availability around them^58^.

The false seedbed technique depletes weed seed banks through mechanical removal of emerged seedlings. Consequently, successful implementation requires precise knowledge of germination-stage water requirements for dominant weed species in the target agricultural system. Where natural precipitation fails to meet these requirements, calibrated irrigation during the post-tillage, pre-sowing interval ensures adequate soil moisture for weed germination synchronization^51^. The X₅₀ parameter, representing the osmotic potential required to inhibit 50% of maximum germination, has been quantified for several weed species in previous studies. Reported values include − 0.22 MPa for *Nicotiana glauca* R. Graham, − 0.33 MPa for *Picnomon acarna*, − 0.41 MPa for *Murdannia nudiflora*, 0.19 MPa for *Trifolium repens* L., − 0.44 MPa for *Bidens pilosa*, and − 0.28 MPa for *Macroptilium lathyroides*. These values reflect interspecific variation in drought tolerance during germination, with more negative X₅₀ values indicating greater resilience to osmotic stress. In this context, *Urospermum picroides*, with an X₅₀ of − 0.30 MPa, exhibits moderate sensitivity to water deficit compared to other weed species^62–64,69−71^.

### Effect of salinity stress on seed germination

**Fig. 6:**
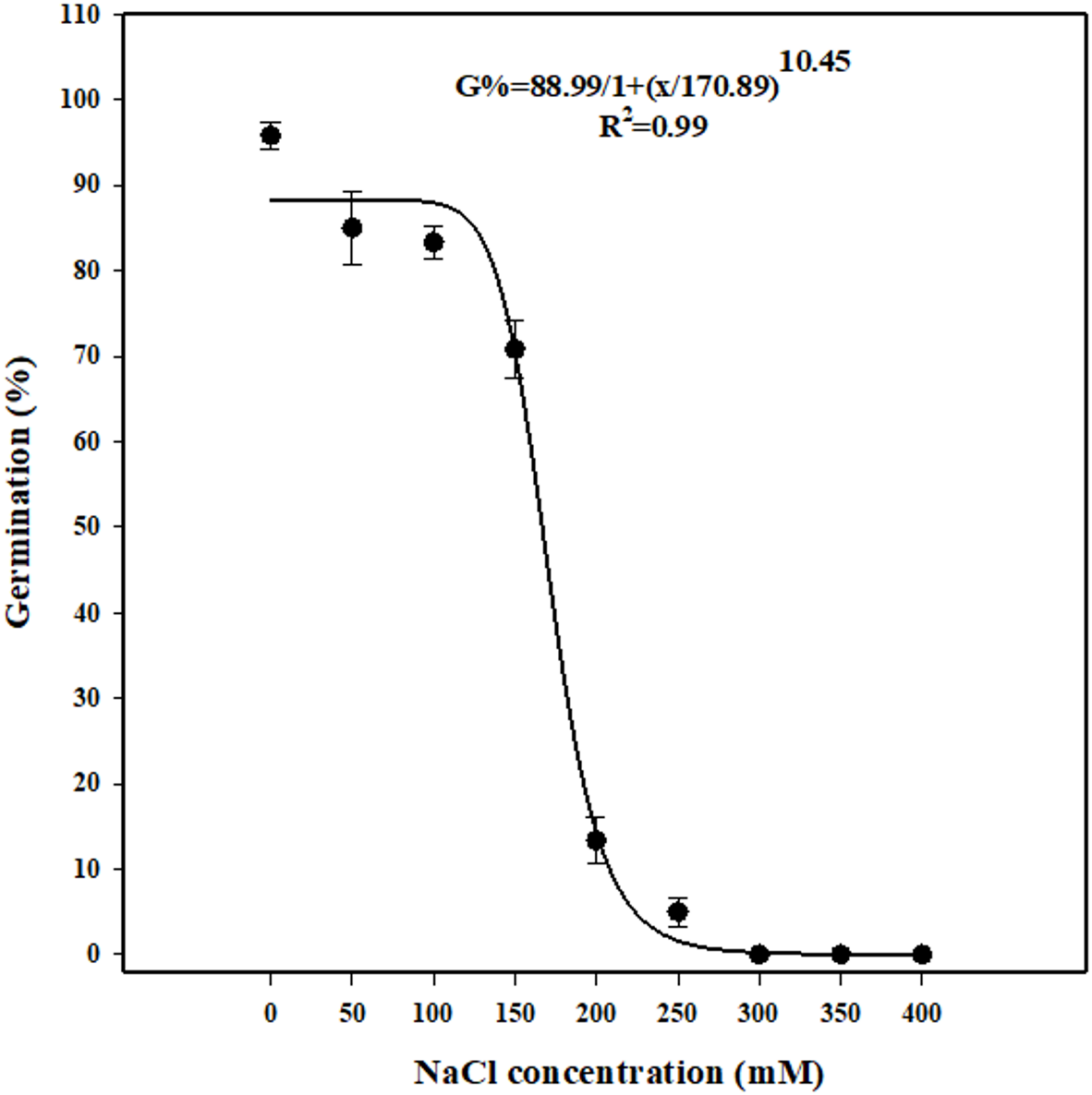
Effect of NaCl concentration on germination of *Urospermum picroides*. Vertical bars represent the mean standard error.

seed germination of *U. picroides* progressively decreased as NaCl concentration increased (Fig. 6). Seeds in the control treatment (0 mM NaCl) exhibited the highest seed germination (95%). Germination decreased to 85%, 83%, and 70% at NaCl concentrations of 50, 100, and 150 mM, respectively. Complete inhibition of germination occurred at concentrations exceeding 300 mM NaCl. At 200 mM NaCl, germination dropped sharply to 13%, and only 5% of seeds remained viable at 250 mM. The NaCl concentration required to reduce maximum germination by 50% was estimated at 170 mM. The reduction in germination percentage was more pronounced at concentrations above 150 mM compared to those below 100 mM NaCl, indicating a threshold beyond which salinity stress severely impairs seed viability and germination capacity (Fig. 6).

The findings of the salinity experiment indicate that *U. picroides* retains germination capacity at salt concentrations ranging from 100 to 200 mM, supporting its potential to establish in saline fields, landscapes, and urban green spaces in Iran, particularly in Khuzestan, where saline soils are widespread and pose significant agronomic challenges. The presence of *U. picroides* in such habitats may exacerbate weed contamination and complicate management strategies due to its resilience under moderate salinity stress. Compared to non-saline conditions, seed germination reductions at 50, 100, 150, 200, and 250 mM were recorded as 11%, 13%, 26%, 86%, and 95%, respectively. These findings suggest that *U. picroides* may act as a problematic weed in saline ecosystems, with implications for crop competition and soil rehabilitation efforts.

Water is essential for hydrating seed tissues, activating respiratory metabolism, and liberating energy and nutrients required for sustained embryo growth. Elevated soil salinity reduces water potential osmotically, thereby impeding cellular water uptake. Consequently, diminished germination capacity arises from impaired reserve mobilization, suppressed synthesis and activity of hydrolytic enzymes, or disruption of cellular turgor^49^.

Ionic toxicity, increased ion diffusion, and elevated salinity levels in plants disrupt metabolic processes, resulting in diminished or inhibited seed germination^72^. Moreover, high salinity deteriorates soil structure and fertility by displacing essential cations such as calcium and magnesium during ion exchange processes^73^. Salinity stress also suppresses the biosynthesis of gibberellic acid, a critical phytohormone for breaking seed dormancy^74^ and significantly impairs water uptake efficiency^75^.

Experimental assessments of salinity tolerance have quantified the X₅₀ parameter, the NaCl concentration required to reduce maximum germination by 50% for several weed species. Reported values include 197 mM for *Mimosa pudica*, 112 mM for *Chamaesyce maculata*, 193 mM for *Eclipta prostrata*, 149 mM for *Macroptilium lathyroides*, 120 mM for *Bidens pilosa*, and 160 mM for *Solanum melanocerasum*. For *Polygonum perfoliatum*, X₅₀ values ranged from 183 to 226 mM across three distinct populations, while *Rhynchosia capitata* exhibited an X₅₀ of 137 mM^19,59,60,70,76–78^. These interspecific differences reflect varying degrees of salinity tolerance during germination, with higher X₅₀ values indicating greater resilience to salt stress. In this context, *U. picroides*, with an X₅₀ of 170 mM, demonstrates high tolerance relative to other weed species, supporting its potential to establish in salt-affected environments.

### Effect of seed burial depth on seedling emergence

**Fig. 7:**
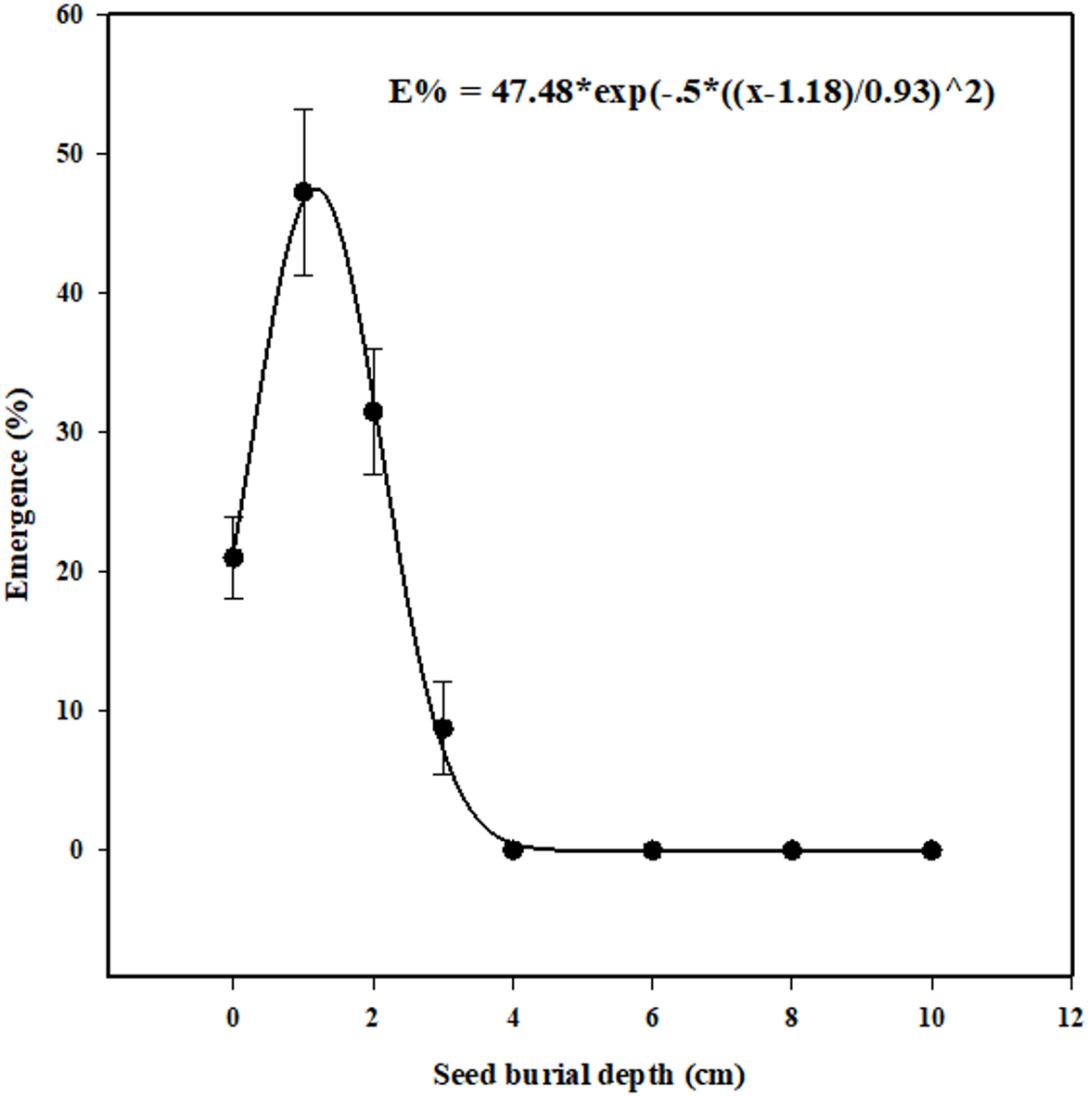
Effect of seed burial depth in the soil on seedling emergence of *Urospermum picroides* 21 days after sowing. Vertical bars represent the mean standard error.

Seed burial depth had a significant impact on the seedling emergence of *U. picroides*. Contrary to expectations of maximum emergence from surface-sown seeds, the highest emergence (47%) was recorded at a burial depth of 1 cm. Seeds placed on the soil surface exhibited limited emergence (21%), likely due to desiccation or insufficient soil contact. Emergence declined progressively with increasing burial depth, reaching 31% at 2 cm and only 8% at 3 cm. While planting seeds at a depth of 1 cm significantly enhanced seedling emergence, deeper burial at 2 and 3 cm led to a progressive decline (Fig. 7). A three-parameter Gaussian model fitted to the emergence data estimated the maximum seedling emergence at 47%, occurring at an optimal burial depth of approximately 1.18 cm. This model effectively captured the unimodal response curve, reflecting the balance between improved seed-soil contact at shallow depths and mechanical constraints imposed by deeper burial.

The reduced seedling emergence observed at the soil surface is primarily due to inadequate seed-soil contact^79^. Seedling emergence of *U. picroides* was significantly lower when seeds were planted on the soil surface compared to buried depths of 1 and 2 cm, suggesting that light is not a limiting factor for germination. Seeds placed on the soil surface exhibit lower emergence than those buried at optimal depths, primarily due to limited water uptake and inadequate seed imbibition^80^. The reduced seedling emergence observed at greater burial depths is likely attributable to limited energy reserves, which are insufficient to support hypocotyl elongation and enable successful emergence from the soil^30,81,82^. Research by^83^ demonstrated that seedling emergence was lower for seeds placed on the soil surface compared to those buried at a depth of 1 cm. Proper inversion tillage (> 4 cm) may be the most effective approach to suppress emergence and deplete soil seed bank reserves.

Several weed species have exhibited higher seedling emergence when buried at depths of 1 to 2 cm compared to placement on the soil surface, including *Sinapis arvensis*^84^ *Hibiscus tridactylites*^85^*Lolium rigidum*^86^*Nassella trichotoma*^83^*Rapistrum rugosum* (L.) All.)^87^*Sicyos angulatus*^88^and *Sesbania cannabina*^89^.

## Conclusion

This study underscores that *U. picroides* seeds exhibit physiological dormancy following rainfall on the parent plants, as evidenced by significant germination enhancement upon gibberellic acid treatment. The highest seed germination was recorded at a constant temperature of 10 °C and alternating temperature 20/10 C. The seeds demonstrated higher germination under saline than drought conditions, indicating greater tolerance to salinity. These findings confirm that *U. picroides* thrives in irrigated environments, while its presence diminishes in fields characterized by higher soil salinity and drought. Cutting timing plays a critical role in influencing seed quality, with mature seeds exhibiting higher seed germination and shallow physiological dormancy. Cutting and mowing during the closed capitula stages, marked by white and yellow achenes, could effectively reduce the weed’s population in subsequent years. Moreover, seedling emergence was significantly higher at shallow burial depths (≤ 1 cm) than at deeper ones, suggesting that mouldboard plowing may reduce the *U. picroides* seed bank, whereas no-till systems encourage its proliferation. Based on recent findings, integrated weed management strategies can be improved by modifying planting dates particularly for urban green spaces and using false or stale seedbeds to reduce the weed seed bank in contaminated areas.

## Materials and methods

### Seed collection, germination test, and storage protocol

Fresh brown achenes of *U. picroides* were collected in May 2020 from open capitula of plants growing as weeds in green areas of the Agricultural Sciences and Natural Resources University of Khuzestan, Bavi, Mollasani, Iran )in coordination with the University Vice Presidency for Research(. The capitula were stored at room temperature (25 °C) in the Weed Science laboratory until completely dried. A seed dormancy-breaking experiment was initiated 10 days post-harvest, using 30 seeds per Petri dish with four replicates. An initial germination test was conducted in an incubator under controlled light and temperature (25 °C) conditions. Seed Germination were below 20% (data not shown), indicating that the seeds possess endogenous dormancy (ED).

### Protocol for germination tests

Petri dishes were sterilized in an autoclave prior to each experiment to eliminate fungal contamination. Thirty seeds of *U. picroides* were placed in each 9-cm-diameter Petri dish on two layers of Whatman No. 1 filter paper, moistened with 5.0 mL of distilled water, polyethylene glycol, or salt concentrations corresponding to the experimental treatments. To prevent evaporation, the Petri dishes were sealed in plastic bags and placed in a growth chamber maintained at a constant temperature of 25 °C with a 12/12-hour light/dark cycle for 21 days. A photosynthetic photon flux density (PPFD) of 105 µmol·m^−2^·s^−1^ was maintained within germination chambers using fluorescent lamps. Germination was assessed based on radicle emergence (≥ 2 mm), which was used as the primary criterion for germination.

### Effect of GA_3_ on breaking seed dormancy

For breaking seed dormancy of *U. picroides*, six concentrations of gibberellic acid (0, 200, 400, 600, 800, and 1000 ppm) were tested under two seed soaking durations (12 and 24 h). Gibberellic acid solutions were prepared at the designated concentrations, and seeds were subsequently placed in flasks corresponding to each concentration. After soaking for the specified durations, seeds were removed from the gibberellic acid solutions, rinsed thoroughly three times with distilled water, and transferred to Petri dishes to initiate the germination experiment.

### Effect of constant, alternating temperatures and light on seed germination

Seed germination was evaluated under eight constant temperatures (5, 10, 15, 20, 25, 30, 35, and 40 °C), three alternating temperature regimes (20/10, 25/15, and 30/20°C day/night), and two light conditions (light/dark and continuous darkness) by incubating 30 seeds in Petri dishes. To simulate natural light conditions under moderate canopy cover and create a realistic germination environment, an additional light source was used to provide a photosynthetic photon flux density (PPFD) of 105 µmol m^−2^ s^−1^. For dark treatments, Petri dishes were wrapped in aluminum foil to block light exposure. To maintain complete darkness durnig the 24-hour dark treatment, seeds were completely shielded from white light, and Petri dishes were examined exclusively under a green safe light .

### Effect of cutting times on seed germination

The capitula of U. picroides were collected from plants growning in the green areas of the Agricultural Sciences and Natural Resources University of Khuzestan, Iran. After harvesting, the capitula were transferred to the laboratory and categorized into five maturation stages based on the openness (open or closed) of the capitula and the color of the achenes (white, yellow, greenish-brown, or brown): (A) closed capitula with white achenes, (B) closed capitula with yellow achenes, (C) closed capitula with greenish-brown achenes, (D) closed capitula with brown achenes, and (E) open capitula with brown achenes (Fig. 4). The capitula were air-dried at room temperature (25 °C) for one week. The dried seeds were then stored at room temperature for six months prior to use in the experiment.

### Effect of osmotic potential on seed germination

Drought stress was simulated using osmotic potentials of 0 (control), −0.1, −0.2, −0.3, −0.4, −0.5, −0.6, −0.8, −1, and − 1.2 MPa, prepared by dissolving polyethylene glycol (PEG) 6000 in distilled water to create the respective osmotic solutions. The experiment was carried out at 25 °C, serving as the reference temperature for preparing PEG 6000 concentrations^90^.

### Effect of salinity stress on seed germination

The effect of salinity stress on seed germination was evaluated using sodium chloride (NaCl) concentrations of 0, 50, 100, 150, 200, 250, 300, 350, and 400 mM. To ensure uniform moisture conditions, 5 mL of the corresponding saline solution was initially added to each Petri dish. Each dish contained thirty seeds and was incubated at 25 °C.

### Effect of seed burial depth on seedling emergence

To assess the impact of sowing depth on seedling emergence, thirty *U. picroides* seeds were sown at various burial depths in plastic pots (10 cm in diameter). Eight burial depths were tested: 0 (soil surface), 1, 2, 3, 4, 6, 8, and 10 cm. The pots were filled with field-collected silty clay soil (40% clay, 41% silt, 19% sand), sourced from the same location as the seed collection site. After sowing, the pots were placed in an incubator maintained at 25 °C under a 12-hour light/12-hour dark photoperiod and a photosynthetic photon flux density of 105 µmol m^−2^ s^−1^.

Watering was performed as needed, ensuring that soil moisture did not exceed field capacity. Seedling emergence was monitored daily for 21 days. Seedling emergence was defined as the visibil appearance of the cotyledon. The assay was concluded when no seedlings emerged for at least three consecutive days. The viability of ungerminated seeds was assessed using tetrazolium staining, as previously described^91,92^.

### Statistical analyses

All experiments were conducted twice using a completely randomized design (CRD) with varying numbers of replications, depending on the specific treatment. The effects of temperature and GA_3_ on seed germination and dormancy breaking were evaluated using three replications per treatment. Experiments assessing the effects of osmotic potential, salinity, and seed burial depth on seed germination and seedling emergence were conducted with four replications per treatment. the effect of cutting time on seed germination was tested using five replications. As no significant differences were observed between the two experimental runs, the data were pooled for analysis of variance (ANOVA). A two-way ANOVA was performed to evaluate the interactive effects of light and temperature on seed germination. All other experiments were analyzed using a one-way ANOVA under a completely randomized design (CRD). Significant differences among treatments were determined using the least significant difference (LSD) test at *P* ≤ 0.05.

Regression analysis was performed using SigmaPlot software (version 14) for all experiments except the temperature study. The coefficient of determination (R²) was used to assess the goodness of fit for the selected models. A three-parameter sigmoidal model (Eq. 1) provided the best fit for germination data from the seed dormancy and cutting time treatments,

The model (Eq. 1) is defined as follows.

1 $$\:G={G}_{max}/\left[\right(1+e-(x-{x}_{50})]/{G}_{rate}])$$

where G is the germination (%) at X, G_max_ is the maximum germination (%), X_50_ is the times (day) or concenteration of GA_3_ required to reach 50% of the maximum germination, and G_rate_ indicates the slope.

Germination percentages from both NaCl and osmotic potential experiments were fitted to a three-parameter logistic model using SigmaPlot software (version 14).

The model (Eq. 2) is defined as follows.

2 $$\:G={G}_{max}/[1+(x/{x}_{50})^Grate]$$

where, G is the total germination (%) at NaCl concentration or osmotic potential x, G_max_ is the maximum germination (%), X_50_ is the NaCl concentration or osmotic potential for 50% inhibition of the maximum germination (%), and G_rate_ is the slope.

The effect of burial depth on seedling emergence was described using a three-parameter peak Gaussian model, as follows (Eq. 3).

3 $$\:E\%={E}_{max}\times\:\text{e}\text{x}\text{p}(-0.5\times\:(\frac{(x-{X}_{0}0)}{{E}_{rate}}{)}^{2}$$

where E% indicates the total seedling emergence (%), E_max_ is the maximum seedling emergence (%), E_rate_ represents the slope, and X_0_ is the burial depth at which maximum seedling emergence occurs.

Models were selected using statistical criteria, including the Akaike Information Criterion (AIC) and the Bayesian Information Criterion (BIC). Models with lower AIC and BIC values were preferred, as these indicate a better fit to the data, a widely accepted approach in model selection^93^.

## Data Availability

Due to data protection and participant confidentiality concerns, the datasets generated and/or analyzed during the current study are available from the corresponding author upon reasonable request.
